# Stimulation of Host Bone Marrow Stromal Cells by Sympathetic Nerves Promotes Breast Cancer Bone Metastasis in Mice

**DOI:** 10.1371/journal.pbio.1001363

**Published:** 2012-07-17

**Authors:** J. Preston Campbell, Matthew R. Karolak, Yun Ma, Daniel S. Perrien, S. Kathryn Masood-Campbell, Niki L. Penner, Steve A. Munoz, Andries Zijlstra, Xiangli Yang, Julie A. Sterling, Florent Elefteriou

**Affiliations:** 1 Department of Pharmacology, Vanderbilt University, Nashville, Tennessee, United States of America; 2 Vanderbilt Center for Bone Biology, Vanderbilt University, Nashville, Tennessee, United States of America; 3 Department of Medicine, Division of Clinical Pharmacology, Vanderbilt University, Nashville, Tennessee, United States of America; 4 Department of Orthopaedic Surgery and Rehabilitation, Vanderbilt University, Nashville, Tennessee, United States of America; 5 Vanderbilt University Institute of Imaging Science, Vanderbilt University, Nashville, Tennessee, United States of America; 6 Department of Medicine, Division of Allergy, Pulmonary, and Critical Care Medicine, Vanderbilt University, Nashville, Tennessee, United States of America; 7 Department of Pathology, Microbiology, and Immunology, Vanderbilt University, Nashville, Tennessee, United States of America; 8 Department of Cancer Biology, Vanderbilt University, Nashville, Tennessee, United States of America; 9 Department of Veterans Affairs (VISN 9), Nashville, Tennessee, United States of America; London Research Institute, United Kingdom

## Abstract

Bone and lung metastases are responsible for the majority of deaths in patients with breast cancer. Following treatment of the primary cancer, emotional and psychosocial factors within this population precipitate time to recurrence and death, however the underlying mechanism(s) remain unclear. Using a mouse model of bone metastasis, we provide experimental evidence that activation of the sympathetic nervous system, which is one of many pathophysiological consequences of severe stress and depression, promotes MDA-231 breast cancer cell colonization of bone via a neurohormonal effect on the host bone marrow stroma. We demonstrate that induction of RANKL expression in bone marrow osteoblasts, following β2AR stimulation, increases the migration of metastatic MDA-231 cells in vitro, independently of SDF1-CXCR4 signaling. We also show that the stimulatory effect of endogenous (chronic stress) or pharmacologic sympathetic activation on breast cancer bone metastasis in vivo can be blocked with the β-blocker propranolol, and by knockdown of RANK expression in MDA-231 cells. These findings indicate that RANKL promotes breast cancer cell metastasis to bone via its pro-migratory effect on breast cancer cells, independently of its effect on bone turnover. The emerging clinical implication, supported by recent epidemiological studies, is that βAR-blockers and drugs interfering with RANKL signaling, such as Denosumab, could increase patient survival if used as adjuvant therapy to inhibit both the early colonization of bone by metastatic breast cancer cells and the initiation of the “vicious cycle” of bone destruction induced by these cells.

## Abstract 2

The activation of sympathetic nerves by psychosocial stress creates a favorable environment in bone for the establishment of cancer cells in a mouse model of breast cancer.

## Author Summary

Improved detection programs and better drugs to eradicate breast tumors have increased survival in women with breast cancer. However, pain and metastasis to distant organs, including bone, remain significant clinical problems. Understanding why and how metastatic cancer cells colonize specific organs is therefore critical if we are to further improve morbidity and mortality for these patients. Using a mouse model of breast cancer bone metastasis, we present evidence that activation of sympathetic nerves, which is typical in chronic stress or depression, promotes the colonization and establishment of metastatic cancer cells within the bone marrow, leading to an increase in bone osteolytic lesions. We show that this effect is mediated via a β-adrenergic receptor-dependent response of the host bone marrow stroma to catecholamines, that are released upon sympathetic activation, and via the pro-migratory activity of RANKL, a cytokine that is well known to promote bone resorption. Of importance clinically, blocking sympathetic activation with a β-blocker, or blocking RANKL signaling in cancer cells, inhibited the stimulatory effect of sympathetic activation on bone metastasis in this mouse model. Stress-induced sympathetic activation may thus explain, at least in part, the reduced survival rate of breast cancer patients experiencing severe depression. The data also support the use of β-blockers or RANKL blockade as possible adjuvant therapy for women with breast cancer.

## Introduction

Breast cancer metastasizes to bone, lung, liver, brain, and lymph nodes. Among these metastases, those targeted to bone are preponderant and observed in approximately 70% of breast cancer fatalities [1]. They are predominately osteolytic and responsible for virtually all breast cancer deaths [2]. Currently available treatments are unable to eradicate metastatic cancer [3] and are limited to the treatment of bone symptoms and complicating fractures. There is thus a critical need for identification of therapeutics that curtail the metastatic process.

The process of cancer metastasis is multifactorial, influenced by a combination of genes [4], and dependent upon intrinsic cancer cell characteristics that dictate how cells migrate, survive, and proliferate, as well as on the cellular and cytokine profile of the tissue from which the cells initially egress. This process is also driven by the microenvironment to which metastatic cancer cells ultimately home [5]. The mechanisms underlying the organ-specific nature of bone metastasis are governed by chemoattractants (e.g. CXCL12/SDF1), attachment molecules (e.g. ALCAM, annexin II), and cytokines regulating cell growth and angiogenesis (e.g. IL6 and VEGF) [6],[7]; however, the conditions and factors that regulate the expression or activity of these critical molecules to affect metastatic cancer cell bone colonization, establishment, tumor growth, metastatic progression, and recurrence remain unclear. Characterization of such is critical, not only to understanding why some patients are more prone than others to bone metastasis or relapse following treatment of the primary cancer, but also for the design of therapeutic interventions to prevent metastasis to distant organs.

The bone microenvironment is a dynamic compartment in which bone is continuously remodeled for proper maintenance of skeletal properties and calcium serum levels, and where hematopoiesis takes place. Thus, it is richly vascularized, but also abundantly innervated by sympathetic, sensory, and glutaminergic nerves [8]. Sympathetic neurons are found in the bone marrow and within cortical bone, and it has become clear during the last decade that they significantly affect both the mesenchymal and hematopoietic lineages that constitute the bone marrow. Norepinephrine (NE)-releasing sympathetic nerves, activated by brainstem and hypothalamic centers, stimulate the formation of osteoclasts, thus favoring bone resorption [8]–[12]. In addition, sympathetic nerves inhibit osteoblast proliferation and regulate hematopoietic stem cell (HSC) proliferation, survival, and trafficking [13],[14]. The osteoblastic niche and the β2 adrenergic receptor (β2AR) appear to be central and necessary mediators of such sympathetic-driven skeletal processes, generating cytokines that play pivotal roles in stimulating osteoclast formation and hematopoietic cell trafficking, including SDF1 [15],[16] and RANKL (receptor activator of NFκB ligand) [17],[18]. Beyond the demonstration that many aspects of skeletal biology are under the control of neuroendocrine factors, these findings suggest that drugs and emotional or pathophysiological conditions that affect sympathetic outflow may influence bone biology and contribute to bone pathologies.

Sympathetic activation is triggered by prolonged or severe emotional stress. It does not affect overall tumor incidence, yet is associated with shorter patient survival and increased recurrence of breast cancer, which suggests that sympathetic activation may contribute to breast cancer metastasis [19],[20]. This hypothesis is supported by mouse models that reveal a linkage of neuroendocrine signaling to increased tumor vascularization, invasiveness, and metastasis in soft tissues, via a direct effect on ovarian or breast tumor cells [21],[22]. The effects of sympathetic activation on bone homeostasis, however, suggest that emotional stress could also indirectly control the behavior of metastatic cancer cells by acting on bone marrow stromal cells, particularly β2AR-expressing osteoblasts. The observation that sympathetic activation increases bone remodeling and bone marrow HSC trafficking via cytokines that are also involved in cancer metastasis further lends credence to this hypothesis. Indeed, SDF1 has clearly been implicated in the mechanisms underlying the homing of metastatic cancer cells, including breast and prostate carcinomas, and RANKL is increasingly recognized as a crucial factor for cancer cell motility, in addition to its well-established role in tumor-induced osteolysis [23].

We show here that sympathetic outflow alters the cytokine profile of the bone microenvironment and promotes the incidence of metastatic colonization by breast cancer cells. We identify RANKL, secreted by osteoblasts in response to sympathetic activation, as a stimulatory factor for breast cancer cell migration and bone colonization in vivo and demonstrate that breast cancer metastasis to bone, induced by increased endogenous sympathetic outflow triggered by restraint stress, can be inhibited by the β-blocker propranolol.

## Results

### Propranolol Blunts the Stimulatory Effect of Endogenous Sympathetic Activation on Breast Cancer Cell Bone Colonization

**Figure 1:**
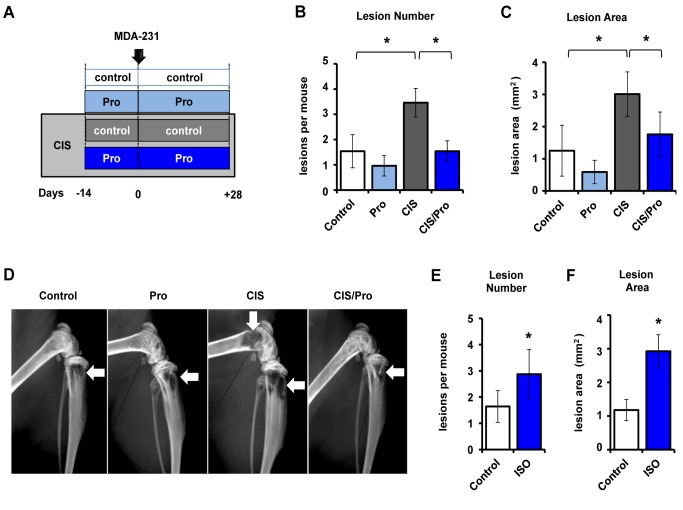
Propranolol inhibits the increase in MDA-231 metastastic bone colonization induced by endogenous sympathetic activation. (A) Mice were given propranolol (Pro) in the presence or absence of 2 h daily chronic immobilization stress (CIS). (B) Bone lesion number and (C) lesion area in the femurs, tibiae, and humeri of nude mice, measured by Faxitron analysis (*n* = 10). (D) Representative Faxitron images of hind limbs 28 d after tumor inoculation, showing osteolytic lesions (white arrows). (E) Number of osteolytic lesions and (F) total osteolytic lesion area/mouse following control versus Isoproterenol (ISO) treatment by Faxitron analysis (*n* = 8). Data are plotted as means ± SEM ; * *p*<.05.

Metastasis to bone is a multistep process starting from the egress of metastatic cells from the primary tumor, contingent upon their subsequent survival in the bloodstream, followed by arrest within the bone capillaries, and colonization of the bone marrow microenvironment. To characterize the effects of sympathetic activation on bone metastasis, we utilized an established model of bone metastasis in which osteotropic GFP-tagged MDA-MB-231VU human mammary carcinoma cells selected at Vanderbilt by in vivo passage of a clone from Dr. T. Yoneda (called MDA-231 cells herein) were inoculated via intracardiac (IC) injection into athymic nude mice [24]. This model is relevant to the late stages of the bone metastasis process, when metastatic cancer cells egress from blood capillaries and reach the bone marrow microenvironment. Chronic immobilization stress (CIS) was chosen as a model of endogenous sympathetic activation (and depression) in rodents [21]. In this experimental paradigm, mice are submitted to bodily restraint for 2 h a day, 6 d a week, for the duration of the trial. Six weeks of CIS treatment, including 2 wk of CIS treatment prior to tumor cell inoculation, induced a significant 2-fold increase in the number of osteolytic lesions (and lesion area), measured by Faxitron, compared to control (no CIS) (Figure 1A–D). Endogenous sympathetic activation also significantly increased bone tumor number (2.5-fold increase), measured by histomorphometry (Figure S1A). In an independent experiment, daily administration of the non-selective β1/β2 adrenergic receptor agonist isoproterenol (ISO, 3 mg/kg i.p.), utilized as a pharmacological surrogate model of sympathetic activation, had a similar stimulatory effect on bone lesion number (Figure 1E), bone lesion area (Figure 1F), and bone tumor number and burden compared to PBS control (Figure S2B–D). The observed increase in bone lesion area induced by CIS and ISO was not surprising considering sympathetic activation is known to promote osteoclastogenesis and bone resorption [8]. The increase in bone lesion and tumor number, on the other hand, supports the hypothesis that sympathetic activation also promotes tumor metastasis and/or growth in bone. Furthermore, the observation that ISO has a similar effect to CIS is evidence that sympathetic activation, rather than activation of the hypothalamic-pituitary-adrenal axis, triggers these effects of CIS on tumor metastasis.

An implication of these findings is that blockade of βAR signaling by β-blockers should inhibit the colonization or growth of metastatic breast cancer cells in bone. To address this question, the β-blocker propranolol was used daily, concomitantly with CIS treatment, to block sympathetic activation induced by CIS. Upon propranolol administration, no significant decrease in bone lesion area and number was observed in control (no CIS) mice. By contrast, in mice subjected to CIS treatment, propranolol significantly decreased the number of osteolytic lesions, their surface, and the number of bone tumors compared to CIS control mice (Figure 1A–C and Figure S1A). These data thus indicate that endogenous sympathetic activation by chronic stress in mice increases the incidence of breast cancer cell metastasis to bone, and that β-blockers have the potential to inhibit this effect, which has obvious clinical implications.

### β2AR Stimulation of Breast Cancer Cells Decreases Extraskeletal Tumor Growth

**Figure 2:**
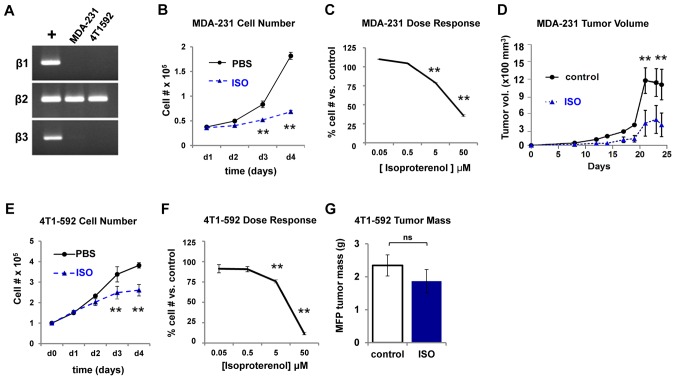
ß2AR stimulation decreases tumor cell growth in vitro **and in vivo.** (A) ß2AR mRNA expression in two bone metastatic mammary carcinoma lines MDA-231 and 4T1-592 assessed by RT-PCR. (B) In vitro effects of ISO (1 µM) on MDA-231 cell number over 4 d (*n* = 3). (C) ISO treatment dose-response on MDA-231 cell number versus control (*n* = 3). (D) Subcutaneous MDA-231 cell growth quantification (tumor volume) following daily PBS (control) or ISO injections (*n* = 6). (E) 4T1-592 cell number in vitro in response to ISO versus control (*n* = 3). (F) ISO treatment dose-response on 4T1-592 cells (*n* = 3). (G) Mammary fat pad tumor mass of 4T1-592 cells in BalbC mice as measured by end point tumor mass after 4 wk of ISO treatment (*n* = 9; *ns*, not significant, *p* = 0.22). Data are plotted as means ± SEM; ** *p*<.005.

Both osteoblasts and breast cancer cells, including MDA-231 and 4T1-592 cells (an osteotropic clone derived from 4T1 cells [25]), express the β2AR (Figure 2A). Therefore, the stimulatory effect of sympathetic activation on breast cancer cell metastasis to bone could be mediated by a direct effect on cancer cells, or by an indirect effect through host bone cells. To assess a possible direct effect of adrenergic stimulation on cancer growth, we first treated MDA-231 and 4T1-592 tumor cells with ISO (1 uM), then measured cell number over a 4-d period in vitro. Surprisingly, ISO decreased MDA-231 and 4T1-592 cell growth over time, in a dose-dependent manner (Figure 2B,C,E,F).

We then used two distinct in vivo models of tumor growth to further assess the direct effect of β2AR stimulation in breast cancer cells. In athymic nude mice inoculated subcutaneously with 10^6^ MDA-231 cells and treated daily with ISO for 3 wk, tumor volume at end point was not increased but rather decreased compared to the PBS control group (Figure 2D). Furthermore, no increase in tumor growth was observed in wild type (WT) BALB/c mice that received mammary fat pad inoculations of 5×10^3^ 4T1-592 cells and subsequent ISO treatment for 4 wk (Figure 2G). These results suggest that direct adrenergic stimulation of breast cancer cells inhibits their growth or promotes cell death in extraskeletal sites, and that the increase in bone tumor lesion and number observed in vivo following CIS treatment is not likely due to the promotion of cell proliferation, but rather to an effect of sympathetic nerves on bone colonization.

### ISO Treatment Increases Tumor Cell Colonization of Bone by Acting on the Host Bone Marrow Environment

**Figure 3:**
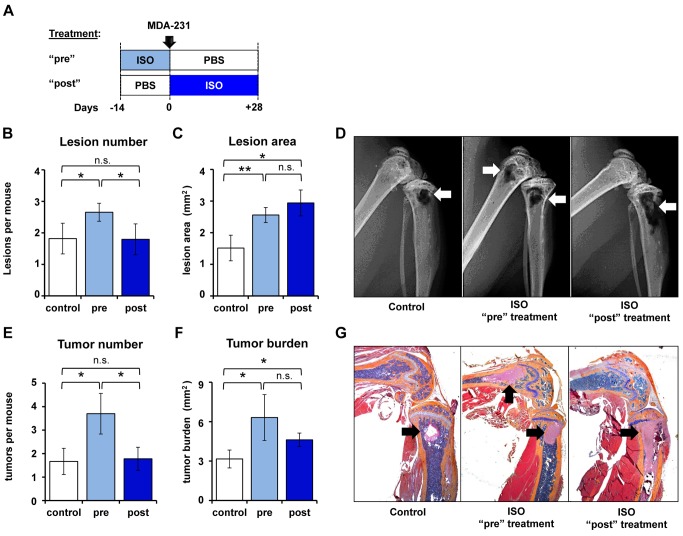
ß2 Adrenoreceptor signaling in bone increases tumor colonization. (A) Athymic nude mice were treated daily with ISO (3 mg/kg) for 2 wk prior to or for 4 wk after intracardiac injection of MDA-231 cells. (B) Number of osteolytic lesions per mouse as quantified from Faxitron radiographs at day 28 in humeri, femurs, and tibiae (*n* = 8). (C) Total osteolytic lesion areas visible per mouse, as assessed by Faxitron (*n* = 8). (D) Representative Faxitron images of hind limbs at day 28. (E) Tumor number counted in femurs and tibiae by histology (*n* = 8). (F) Total bone tumor burden in femurs and tibiae by histology (*n* = 8). (G) MDA-231 tumors appear as pink masses in the metaphyses (modified H&E). Data are plotted as means ± SEM; * *p*<.05; ** *p*<.005.

To further understand the mechanism whereby sympathetic activation promotes breast cancer bone metastasis, we administered ISO to nude mice before MDA-231 cell intracardiac inoculation (“pre”-treatment for 2 wk) or after MDA-231 cell inoculation (“post”-treatment for 4 wk) (Figure 3A). We reasoned that if sympathetic activation promotes cancer cell colonization in bone via an indirect effect on the stroma, treating mice with ISO *prior* to tumor inoculation (with no further treatment afterward) may increase the incidence of tumors in bone and possibly the number of osteolytic lesions, whereas treating mice *after* tumor inoculation should promote bone resorption and increase the area of osteolytic lesions, but not bone colonization.

In tumor bearing bones, ISO treatment for 4 wk post-MDA-231 cell inoculation significantly increased the size, but not the number, of osteolytic lesions, assessed by radiographic analyses (Figure 3A–D). Accordingly, ISO “post”-treatment also increased tumor burden, but not tumor number, as measured by histomorphometry (Figure 3E–G). In contrast, ISO “pre”-treatment increased bone lesion and tumor areas, but also increased bone lesion and tumor numbers. More metastatic tumors and bony lesions were thus formed in the “pre”-ISO treatment group, even though MDA-231 cells were never directly subjected to ISO stimulation, indicating that ISO promotes metastasis to bone via its effect on the bone marrow environment and not the tumor cells themselves. In agreement with studies in euthymic mice, non-tumor-bearing bones from athymic mice treated with ISO for 4 wk displayed a 32% decrease in tibial trabecular bone volume (BV/TV), assessed by 3-D microtomography (uCT) (Figure S2A). The surface of TRAP-positive osteoclasts was significantly increased in these mice (Figure S2B), and accordingly, *Rankl* expression in long bones was increased 17-fold in response to ISO when compared to PBS controls (Figure S2C). Tumor bearing tibiae, on the other hand, displayed a 76% decrease in BV/TV (Figure S2D). Collectively, these results indicate that sympathetic activation alters the bone marrow environment to make it more hospitable for breast cancer cell metastatic colonization, establishment, and growth, thus priming the “vicious” cycle of bone destruction induced by cancer cells, and exacerbating this cycle once tumor burden increases.

### β2AR Signaling in Bone Marrow Osteoblasts Increases the Migration of Mammary Carcinoma Via RANKL

**Figure 4:**
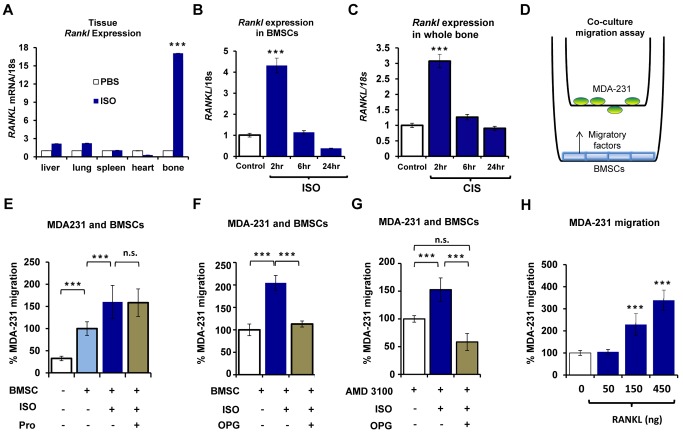
Osteoblast-derived RANKL promotes the migration of MDA-231 cells in response to ISO treatment. (A) Whole bone (tibia and femur, including marrow, excluding growth plate) *Rankl* expression in response to ISO treatment (3 mg/kg) in vivo compared to other soft tissues measured by qPCR (2 h treatment, control PBS versus ISO, *n* = 4). (B) *Rankl* expression in ISO-treated BMSCs measured by qPCR. (C) *Rankl* expression time course in long bones from mice subjected to daily restraint stress for 2 wk (CIS) (*n* = 3). (D) Schematic of the osteoblast-MDA231 co-culture transwell migration assay. (E) Transwell migration assays of MDA-231 cells toward BMSCs (*n* = 3), in the presence of 10 uM ISO (*n* = 3) or Pro (10 uM) treatment (*n* = 2). (F) Transwell migration assays of MDA-231 cells toward BMSCs in the presence of ISO or OPG (1 ug/mL) treatment (*n* = 2). (G) Transwell migration assays of MDA-231 toward BMSCs in the presence of ISO or OPG or AMD 3100 (10 ng/mL) treatment (*n* = 3). (H) MDA-231 transwell migration assay in response to soluble rRANKL (*n* = 3). All in vitro assays repeated at least 2 times. Data are plotted as means ± SEM; *** *p*<0.001.

RANKL is a cytokine well-known for its osteoclastogenic properties and for being a critical mediator of the feed-forward cycle of bone destruction induced by bone metastatic cancer cells. It is also expressed by normal mammary gland epithelial cells, contributing not only to the development of the lactating mammary gland during pregnancy [26] but also to cancer cell migration in melanoma [27]. In agreement with other reports [27],[28], we found that parental non-osteotropic breast carcinoma-derived ATCC MDA-231 cells express *RANK*, the receptor for RANKL, but at a lower level than bone metastatic MDA-231 cells (Figure S3A). In contrast, *RANK* expression was not detected in MCF-7 cells. These results, coupled with the observation that ISO strongly increases the expression of *Rankl* in bones—to a greater extent than in any other organs tested (Figure 4A)—led us to explore whether this cytokine contributes to the effect of sympathetic activation on breast cancer metastasis to bone.

First, as observed in bone in vivo, ISO significantly increased *Rankl* expression in primary bone marrow stromal cells cultured in vitro (BMSCs, Figure 4B) as well as in MC3T3 osteoblasts (Figure S3B), which suggests that among bone marrow cells, the osteoblast lineage represents a main target. Second, endogenous sympathetic activation by CIS, like ISO treatment, significantly increased *Rankl* expression in bone 2 h post-stimulation in mice that have been subjected to CIS for 2 wk, indicating that the chosen CIS regimen does not lead to desensitization, at least within the early critical period of bone colonization focused on in this study (Figure 4C). Third, BMSC expression of *Sdf1*, a major cytokine promoting breast cancer cell migration, was unaffected by ISO treatment for 2, 6, or 24 h (Figure S3C); in breast cancer cells treated with ISO, the expression of *RANK* and *CXCR4*—the receptors for RANKL and SDF1, respectively—remained unchanged as well (Figure S3D,E).

To functionally demonstrate the role of RANKL in the migration of metastatic breast cancer cells, transwell migration assays were performed. When MDA-231 cells were plated in the transwell filter without osteoblasts in the bottom chamber, ISO treatment did not increase their migratory properties (unpublished data). Contrastingly, in co-culture transwell assays, primary BMSCs plated in the bottom chamber increased the migration of MDA-231 cells plated on the transwell filter, and ISO treatment significantly exacerbated this effect (Figure 4D,E). ISO treatment of osteoblasts, prior to the addition of propranolol-treated MDA-231 cells to the transwell filters (in order to block β2AR signaling specifically in cancer cells), did not inhibit cell migration (Figure 4E), signifying that the effects of ISO on the migration of MDA-231 cells are mediated by β2AR stimulation in osteoblasts. Addition of recombinant OPG, a soluble decoy receptor for RANKL, blocked the effect of ISO on MDA-231 cell migration in this transwell co-culture assay, indicating that RANKL is the main cytokine involved in ISO-mediated stimulation of MDA-231 cell migration toward BMSCs (Figure 4F). Similar results were obtained by co-culture of MDA231 cells with MC3T3 osteoblasts (unpublished data). Inhibition of SDF1-CXCR4 signaling by AMD3100 did not block the ISO-induced increase in MDA-231 cell migration, but further reduced migration when used in combination with OPG (Figure 4G). These data are in agreement with the observation that pharmacological blockade of the SDF1-CXCR4 axis by AMD3100, a CXCR4 antagonist, does not fully prevent bone metastasis [27]. Lastly, recombinant soluble RANKL (rRANKL) dose-dependently stimulated the migration of MDA-231 cells (Figure 4H), and consistent with the above observations, high *RANK*-expressing MDA-231 cells, but not low *RANK*-expressing parental ATCC MDA-231 cells, responded to rRANKL with a significant increase in migration (Figures S3A and S4A). Recombinant soluble RANKL did not affect MDA-231 cell proliferation (Figure S3F). In sum, these results demonstrate that β2AR stimulation in osteoblasts in vitro promotes breast cancer cell migration via RANKL and via an SDF1-independent mechanism.

**Figure 5:**
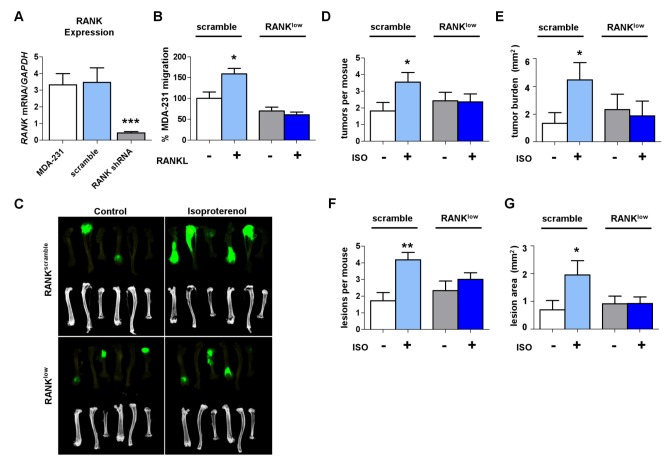
RANK knockdown in MDA-231 cells prevents the increase in bone metastasis induced by ISO treatment. (A) *Rank* mRNA levels in MDA-231 cells and in MDA-231 cells transfected with scrambled non-targeting shRNA vector or RANK shRNA (*n* = 2). (B) Transwell migration of Rank^scramble^ and Rank^low^ MDA-231 cells in response to soluble RANKL at 200 ng/ml (*n* = 2). (C) Representative images of GFP-positive long bones correlating with osteolytic lesions after 4 wk in ISO-treated mice that were inoculated with Rank^scramble^ or Rank^low^ MDA-231 cells. (D–E) Quantification of GFP^+^ tumor number and tumor burden per mouse from ex vivo Maestro imaging (*n* = 9). (F–G) Number and area of osteolytic lesions visible in Faxitron images at 4 wk (*n* = 9). Data are plotted as means ± SEM; * *p*<.05; ** *p*<.005; *** *p*<.001.

To address whether the pro-migratory activity of RANKL observed in vitro contributes to the stimulatory effect of sympathetic activation in breast cancer metastasis to bone in vivo, we used a loss-of-function strategy to specifically reduce RANK expression in metastatic MDA-231 cells. The advantage of this strategy, compared to using a RANKL blocker like OPG, was that it allowed us to assess whether sympathetic activation promotes breast cancer bone colonization via the pro-migratory effect of RANKL on metastatic cancer cells or via an indirect stimulatory effect on bone turnover, since sympathetic activation increases bone remodeling, potentially increasing the expression, activity, and/or availability of other cell- or ECM-derived cytokines promoting cancer cell bone colonization, establishment, and growth. A shRNA knockdown approach was used to generate stable clones of MDA-231 cells expressing reduced levels of RANK. We selected a clone whose *RANK* expression was decreased by 85% (RANK^low^) compared to scramble shRNA control (RANK^scramble^) (Figure 5A). No difference in cell proliferation or *PTHrP* expression was detected between MDA-231 RANK^scramble^ control cells and RANK^low^ cells, treated or not with rRANKL (Figure S4B,C). In contrast, RANK knockdown significantly reduced MDA-231 cell migration in response to rRANKL in a transwell assay (Figure 5B), demonstrating the necessity of a functional RANKL-RANK signaling pathway for the migratory properties of MDA-231 cells. We then inoculated MDA-231 RANK^scramble^ control cells or RANK^low^ cells intracardially to nude mice treated daily with ISO for 6 wk to mimic sympathetic activation. Confirming the first set of results (Figure 1), when control MDA-231 RANK^scramble^ cells were used, ISO treatment significantly increased the incidence of GFP-positive tumors in bone, the number and area of bone lesions measured by Faxitron, and bone tumor burden measured by ex vivo GFP imaging, when compared to PBS-treated mice (Figure 5C–G). In contrast, selective inhibition of RANK expression in MDA-231 RANK^low^ cells blunted the effect of ISO on each of these parameters. No significant difference was observed between the two clones in absence of ISO treatment. These results demonstrate that RANK expression in breast cancer MDA-231 cells, independently of increased bone turnover, is required for their migratory response toward RANKL-expressing bone cells in vivo in response to sympathetic activation.

## Discussion

Although the genetic and phenotypic make-up of a tumor determines its metastatic efficiency, a receptive microenvironment is a prerequisite for tumor colonization, establishment, and growth in distant sites. In the case of breast cancer, the interaction of cancer cells with the bone microenvironment is crucial for their preferential colonization of bone, as well as their subsequent survival, growth, and osteolysis-promoting activity [23]. Characterization of the conditions and factors that transform the bone microenvironment to a state more favorable for cancer cell colonization, dormancy escape, and growth is thus of great interest. In this study, we show that activation of the sympathetic nervous system, a hallmark of severe stress and depression, promotes breast cancer cell colonization in bone. We demonstrate that this neuronal effect on bone metastasis is mediated via the β2AR in bone-forming cells of the host bone marrow environment, and not by a direct effect on metastatic cancer cells, and furthermore that RANKL, whose expression is induced in osteoblasts by sympathetic activation, mediates this effect in vitro and in vivo via its pro-migratory activity. Additionally and of clinical importance, we show that RANKL signaling and the β-blocker propranolol can inhibit the stimulatory effect of endogenous sympathetic activation on breast cancer bone metastasis.

Sympathetic nerves releasing NE are present within bone in the vicinity of bone cells, and the β2AR is broadly expressed in bone cells of the mesenchymal, monocytic, or immune lineages, as well as in several cancer cell lines, including mammary carcinomas [27]. Additionally, epinephrine released into the circulation following stress is also an agonist for the β2AR. Sympathetic activation can thus directly stimulate the β2AR in metastatic cancer cells to promote their survival during anoikis, their colonization of bone, and their growth following bone establishment. Such direct stimulatory mechanisms have been reported for ovarian [29] and prostate [30] cancer cell lines. A stimulatory effect of chronic stress has been observed as well on the in vivo growth of parental (non-osteotropic) MDA-MB-231 breast cancer cells implanted orthotopically in the mammary fat pads (MFP) [21]. In contrast, however, β2AR stimulation of the bone metastatic MDA-231 clone, which is derived from MDA-MB-231 cells following in vivo selection of bone osteotropic cells [31], reduced tumor growth when cells were implanted subcutaneously. Catecholamines may thus promote or restrain breast cancer cell proliferation depending on either the site of cell growth or the genetic make-up of each cancer clone. On the other hand, the results of our study indicate that β2AR activation in host stromal osteoblasts predominantly accounts for the stimulatory effect of sympathetic activation on MDA-231 breast cancer cell bone colonization. This is supported by the observation that cancer cell inoculation in mice pre-treated for 2 wk with ISO can increase the number of bone tumors and lesions. In that experimental setting, cancer cells are not directly subjected to ISO stimulation; therefore the stimulatory effect of ISO on breast cancer cell bone metastasis must occur via stimulation of the β2AR in host stromal cells, and not via a direct effect on breast cancer cells. It is further reinforced by the observation that selective deletion of the β2AR in osteoblasts recapitulates the high bone mass induced by global β2AR deletion [32], suggesting that β2AR signaling in osteoblasts, and not vascular cells or immune cells for instance, contributes to the regulation of bone remodeling and of the bone marrow cytokine profile. It is also of note that the increase in RANKL expression induced by ISO was more pronounced in the MC3T3 osteoblastic cell line when compared to BMSCs, suggesting that among the adherent stromal cells constituting the bone marrow, osteoblasts represent the main target for the effect of sympathetic nerves on RANKL expression and breast cancer cell bone colonization. Of interest is that osteocytes also secrete RANKL [33] and thus might also represent a critical bone mesenchymal cell population associated with the aforementioned sympathetic effect. Lastly, the absence of effect of recombinant RANKL on MDA-231 cell proliferation reinforces the notion that the release of RANKL by bone marrow osteoblasts promotes the colonization or retention of metastatic cancer cells, and not tumor growth. It thus appears that sympathetic activation has significant effects on both the host bone marrow stroma and metastatic cancer cells, and that the direct effect of β2AR stimulation on cancer cells depends on the intrinsic characteristics of these cells and on their tissue localization. The observation that the inhibitory effect of ISO on MDA-231 cancer cell proliferation was prevented by coculture with bone marrow osteoblasts (and not fibroblasts) further supports the importance of the tumor microenvironment on the growth of various metastatic cancer cell clones (Figure S5).

Stress and severe depression activate both sympathetic outflow and the HPA axis, and thus the potential role of HPA activation in the behavior of metastatic cancer cells in CIS-treated mice cannot be excluded. The observation that bone metastasis induced by CIS could be replicated by βAR stimulation and be blocked by a selective βAR antagonist like propranol strongly suggests that the contribution of adrenergic signaling is very significant and possibly predominant in this process. It is possible that the stimulatory action of glucocorticoids on β2AR signaling in osteoblasts [34] could in fact augment adrenergic signaling in this cell lineage to promote bone metastasis following CIS-induced sympathetic and HPA axis co-activation. This putative mechanism may have important implications for the effects of exogenous glucocorticoids given in conjunction to chemotherapy to cancer patients.

The use of adrenergic agonists in our experimental strategy raised the possibility that sympathetic activation could influence breast cancer bone colonization by a stimulatory effect on blood flow or angiogenesis, as observed in other types of cancers [35]–[37], which would lead to higher likelihood of metastatic cell engraftment. The fact that mice were taken off treatment (ISO or CIS) 24 h before intracardiac injection of cancer cells and that cardiovascular parameters returned to normal by 2–3 h following ISO injection in nude mice, as measured by Visen ultrasound studies (Figure S6A–F), exclude, however, a contribution of blood flow on this effect of sympathetic activation on tumor metastasis to bone. Immune cells may be involved as well since they respond to sympathetic signals, but the demonstration of an effect of sympathetic activation on breast cancer bone metastasis in immunodeficient nude mice precludes any contribution of T cells to this model. Lastly, the evidence that sympathetic activation promotes breast cancer cell metastasis to bone via a host-mediated mechanism suggests that such pathophysiological conditions influence other types of osteotropic solid tumors, which warrants further research.

RANKL is well known for its osteoclastogenic properties during typical bone turnover. The importance of RANKL in promoting tumor growth in bone and osteolysis induced by metastatic breast cancer cells is also well established [38] and has led to the use of RANKL blockade to prevent fracture in patients with breast and prostate cancer [39]. Over the last few years, it has become increasingly recognized that this cytokine plays a broader role in the process of cancer cell metastasis [40]. The protective effect of RANKL blockade by recombinant OPG on melanoma bone metastasis reported by Jones and collaborators was the first to suggest that RANKL, by enhancing the migration of B16F10 melanoma cancer cells, was implicated in promoting metastatic bone colonization and establishment. In our study, the finding that RANK knockdown in metastatic breast cancer cells reduces bone metastasis indicates that this signaling axis is used by metastatic breast cancer cells as well for their bone metastatic potential, independently of the increased bone turnover induced by sympathetic activation. These findings differ from the Jones study in that this effect was only observed in mice subjected to β2AR stimulation (and high levels of RANKL in bone), and not in non-challenged mice. Regardless of the pathophysiological factor(s) increasing its expression or activity, our findings indicate that RANKL is one of the important “soil” factors that promote the colonization of bone by breast cancer cells. Whether RANKL promotes the recruitment of circulating metastatic breast cancer cells from the circulation into bone, or the retention of these cells within the bone marrow environment following egress, remains to be determined. The links encompassing severe depression, high sympathetic tone, increased RANKL expression in bone, and higher incidence of bone metastasis reported in our study, as well as the poor prognosis of breast cancer patients with high tumor RANK expression [41], provide an explanation for the observed correlation between emotional stress and reduced survival of patients with breast cancers [42]–[44], which will obviously have to be confirmed by further clinical or epidemiological studies.

Because PTHrP and other cytokines converge on the RANKL pathway once tumor load increases, the effect of sympathetic activation on RANKL expression by host bone marrow osteoblasts is likely to be most relevant to the early phases of bone colonization by metastatic cancer cells and to the initial steps of the osteolytic cycle of bone destruction induced by metastatic cancer cells, when bone tumor burden is still low. The derived therapeutic implication is that drugs targeting RANKL, including Denosumab (which is currently only approved for the prevention of bone fracture in patients with breast and prostate cancer), could be efficacious not only to treat the bone symptoms associated with bone cancer metastasis, but also to prevent or limit the development of the disease itself, by reducing breast cancer bone metastasis and/or the activation of dormant breast cancer cells, if administered as adjuvant therapy. The observation that blockade of RANKL in prostate cancer patients increases metastasis-free survival according to a recently published clinical trial supports this prediction [45]. The use of β-blockers may have similar effects to RANKL blockade and could represent an alternative to the current standard of care, with a possible milder effect on bone turnover and a proven safety profile. This is compelling when considering preventative treatments and thus long-term use, and when taking into account treatment cost. Importantly, three recent studies [42]–[44] reported a beneficial effect of β-blocker drug therapy on secondary breast cancer formation and patient survival. These clinical studies support the contribution of sympathetic signaling to the bone metastatic process, and the use of β-blockers as possible adjuvant therapy for breast cancer patients, especially in combination with chemotherapy [46]. Prospective clinical trials will be needed to ascertain the efficacy of β-blockers or RANKL blockers to increase survival in breast cancer patients. If successful, this clinical translation may impact the treatment of millions of women word-wide with an alternative cost-effective treatment.

## Materials and Methods

### Animal Models

All procedures were approved by the Institutional Animal Care and Use Committee at Vanderbilt University Medical Center. Mice were group housed in plastic cages (*n* = 5/cage) under standard laboratory conditions with a 12-h dark, 12-h light cycle, a constant temperature of 20°C, and humidity of 48%. Mice were fed a standard rodent diet (Pharma Serv, Purina Rodent Laboratory Chow 5001; Framingham, MA). Nude mice were housed in sterile conditions and fed autoclaved standard chow. Isoproterenol (ISO) treatment was given as daily intraperitoneal injections (3 mg/kg in 100 uL sterile PBS). Control mice were not given injections. Propranolol groups received propranolol *ad libitum* (0.5 g/L) via drinking water. Chronic Immobilization Stress (CIS) was carried out by placing mice in 50 mL laboratory conical tubes, perforated for adequate air supply, for 2 h daily.

### MDA-231 Intracardiac Bone Metastasis Model

MDA-231VU used in this study were derived from MDA-231SA, a highly bone metastatic GFP-tagged clone developed by Dr. T. Yoneda by in vivo passage via intracardiac injection and subsequent culture of tibial metastases. MDA-231VU cells were then FACS sorted for GFP to enrich for GFP-positive cells. MDA-231VU cells were cultured in 10% FBS DMEM with 1% penicillin streptomycin. Cells were trypsinized at 70%–90% confluence, rinsed, and re-suspended in cold PBS at 10^^7^ cells/mL. Athymic nude Foxn1^nu^ female mice aged 4–6 wk were anesthetized and injected in the left cardiac ventricle with 100 uL of cell suspension (10^6^ MDA-231VU cells). Bone metastasis was assessed weekly for 4 wk with Maestro in vivo fluorescence and Faxitron radiographic imaging. In order to prevent cardiac and blood flow complications, all treatments were stopped at least 12 h prior to the intracardiac injection procedure.

### In Vivo Tumor Growth Assay

The 4–6-wk-old Foxn1^nu^ BALB/c (nude) mice were inoculated subcutaneously with 100 uL of PBS solution containing 1×10^6^ MDA-MB-231 at the dorsal midline between the scapulae. Measurements of tumor dimensions were made every 2–3 d with calipers or via Maestro GFP imaging. For 4T1-592, 50 uL of a PBS solution containing 5×10^3^ cells were injected into the 4^th^ mammary fat pad. Tumor size was assessed longitudinally with caliper measurements or volume and post-mortem by weighing on a balance.

### Radiographic Analysis

Osteolytic lesions were quantified bilaterally in the humeri, femora, and tibiae at end point from Faxitron images. Presence of tumor within the bones was confirmed with GFP imaging or by histology. Lesion area was calculated as the average of total osteolytic area per mouse (sum of all six bones counted). Lesion number was calculated as total number of long bones with a visible lesion per mouse (e.g. 0/6, 1/6, etc.). Data were double-blinded and calculated by at least two independent researchers.

### Microcomputed Tomography (μCT) Analysis

Tibiae from each animal were dissected, cleaned, and fixed for 48 h in 10% formalin/PBS, transferred to 70% EtOH, then loaded into 12.3-mm-diameter scanning tubes, and imaged (μCT 40; Scanco Medical, Bassersdorf, Switzerland). The scans were integrated into three-dimensional (3-D) voxel images. A Gaussian filter (sigma = 0.8, support = 1) was used to reduce signal noise, and a threshold of 300 was applied to all analyzed scans. Scans were done at 12 µm resolution (E = 55 kVp, I = 145 µA). Two hundred transverse slices of the proximal tibia were taken from the growth plate and extended distally. All trabecular measurements were made by manual determination of appropriate slices to exclude growth plate, and automated contouring using voxel counting and sphere-filling distance transformation indices.

### Histology

Following uCT scanning, bones were decalcified in 20% EDTA pH7.4 at room temperature for 3–4 d. Decalcified samples were then dehydrated and embedded in paraffin. A modified H&E-phloxine-Orange G stain was used to quantify tumor burden and tumor number in 5 um paraffin sections. Metastatic tumor foci were identified by morphology and pink staining in contrast to blue bone marrow cells. Tumor number was counted as number of long bones present with a tumor. Tumor burden was quantified using the Bioquant system imaging software (Nashville, Tennessee) as total tumor area per combined area of the six long bones averaged from three sections per bone. Osteoclasts were visualized and counted following Tartrate Resistant Acid Phosphatase (TRAP) staining using a standard protocol and the Bioquant system.

### Migration Assays

MDA-231VU cells were detached with trypsin and resuspended in 10% FBS for 1 h prior to assay. Cells were plated in serum-free DMEM in the top well of a 96-well Boyden chamber apparatus (Neuroprobe) and allowed to migrate through a semipermeable (8 uM pore size) membrane towards 2.5% FBS DMEM in the bottom chamber for 4–6 h. Co-culture migration assays were conducted with two separate transwell systems: a 24-well format using Corning 8 um pore transwell inserts and a 96-well Boyden chamber (Neuroprobe). Primary BMSCs or MC3T3 osteoblasts were grown to confluence in 24- or 96-well tissue culture plates. 24 h before migration, fresh 2.5% FBS DMEM containing 10 uM isoproterenol or PBS was added to the cells. On the day of migration, semipermeable membranes were added to the plates, on top of which cancer cells were plated in serum-free media. In both types of assays, unmigrated cells were removed with wet kimwipe from the top of the membrane and were then subsequently fixed with 10% PBS buffered formalin and stained with crystal violet. Total cell area per well was then quantified with an area scan on a 96-well plate reader (Synergy2, Biotek).

### Growth Assays

MDA-231VU and 4T1-592 cell growth was assessed in Phenol-free DMEM containing 2.5% serum, in a 96-well format. Medium was changed daily with fresh ISO or PBS. Cell number was quantified daily by GFP signal measurements (MDA-231VU cells) or crystal violet staining and OD_592_ reading after fixation (4T1-592), using a Synergy2 plate reader (Biotek). Growth curves were normalized against day 0. Each treatment contained eight replicates. Dose-response was calculated based on cell number after 4 d of growth. For coculture growth assays, BMSCs, MC3T3, or NIH3T3 cells were grown to confluence in 96-well plates in 10% FBS DMEM. 2.5×10^3^ MDA-231VU cells were then plated onto these cells in Phenol-free DMEM containing 2.5% serum, which was changed daily along with treatment. Total GFP signal per well was quantified with a Syngery2 plate reader. Total counts at each time point were reported as raw numbers or normalized against day 0 reading as indicated.

### Gene Expression Assays

For all gene expression assays, total RNA was extracted from tissues or cells using TriZOL. Tissue RNA extraction was performed following tissue snap-freezing in liquid N_2_ and power generation using a N_2-_chilled mortar and pestle, prior to homogenization in TriZOL. RNA quality and quantity were then checked by spectrophotometer and 28S/18S band integrity on a 2% formaldehyde/agarose denaturing gel. cDNA was generated using the High Capacity Reverse Transcriptase Kit (Applied Biosystems #438814). Real-time PCR was performed using TaqMan gene expression assays on a BioRad CFX96 Real Time System. Taqman probes/primers were from Applied Biosystems [*Adr*β*2*, Mm02524224_s1; CXCR4, Hs00237052_m1; Cxcr4 *Mm01292123_m1*; *Cxcl12 (Sdf1)*, *Mm00445553_m1*; *RANK (TNFRSF1)*, *Hs00187192_m1*; *Rankl*, Mm00441908_m1; *18s* RNA (DQ) MIX probe dye: FAM-MGB, 4352655; *Hprt1*, *Mm00446968_m1*]. Results were analyzed using standard curve quantification or ddCt methods. RT-PCR for βAR1-3 expression was performed as described previously [18].

### ShRNA Knock Down

MDA-231 (1×10^6^) cells were transfected with HuSH-29 (Origene, Rockville, MD) shRNA silencing vectors against human *RANK* (*TNFRSF11A*) 5′GGAAAGCACTCACAGCTAATT3′ or scramble control: 5′GGAATCTCATTCGATGCATAC3′, which are placed behind a U6 promoter. Transfections were carried out with the shRNA Nucleofector Kit V (Lonza, Walkersville, MD) and program X-013 per manufacturer's instructions. Selection with 1 µg/mL puromycin was begun following overnight recovery. Individual colonies were isolated and maintained with 1 µg/mL puromycin. QPCR was performed as described above to evaluate *RANK* knockdown efficiency and verify permanent, stable knockdown over (>5) multiple passages.

### Echocardiography

Cardiac ultrasounds were performed on athymic nude mice before, during, and after IP injection of ISO under isoflurane anesthesia (3% induction 1.5% maintenance). Mice were positioned dorsally on a heated manipulation table and measurements taken with Visualsonics Vevo 770 Ultrasound. Three diameteric measurements of the ascending aorta, closest to the semilunar valve, were obtained in short parasternal axis view, b mode. Three measurements of heart rate were obtained in m mode, using distance between diastolic peaks in the left ventricle. Detailed hemodynamic data were collected at baseline and 24 h after stimulation with ISO (3 mg/kg). Heart rate measurements were repeated at −5 min pre-injection time 0, 1 min post-injection, and then 2, 5, 8, 10, 20, 30, 60, and 240 min post-injection.

### Statistics

All data are presented as means ± SEM. Statistical analyses were performed using one-way ANOVA for multiple comparisons and two-tailed Student's *t* tests, either paired or unpaired with Welch's correction for two-group comparisons. For all analyses, *p*<0.05 was considered significant.

## Supporting Information

Figure S1 ß2AR stimulation increases total number and growth of bone tumors. Quantification of total number of osseous tumors per mouse (A) with chronic Immobilization Stress (CIS) and/or daily propranolol (Pro) (*n* = 10) and total number (B) and size (C) of tumors in bone 28 d after tumor inoculation (*n* = 8). (D) Representative histological images of modified H&E, phloxine, orange G-stained paraffin sections. Tumors (black arrows) are identified by pink staining. Data are plotted as means ± SEM; * *p*<.05; *** *p*<.001.
(TIF)

Figure S2 ß2AR stimulation increases bone resorption in athymic nude mice. (A) Tibial Bone Volume/Tissue Volume (BV/TV) of non-tumor bearing athymic mice treated daily with ISO for 4 wk (*n* = 9). (B) Osteoclast Surface per trabecular Bone Surface (Oc.S/BS) in non-tumor bearing tibiae (*n* = 9). (C) Bone *Rankl* mRNA expression assessed by qPCR (*n* = 4). (D) Tibial BV/TV of tumor-bearing athymic mice treated daily with ISO for 4 wk after intracardiac injection of MDA-231 tumor cells (*n* = 9). Data are plotted as means ± SEM; * *p*<.05; *** *p*<.001.
(TIF)

Figure S3 *Rankl*, but not *Sdf1* expression, is increased in osteoblasts upon ß2AR stimulation. (A) *RANK* mRNA expression in the MDA-231 bone metastatic sub-clone compared to MCF-7 and parental MDA-MB-231 (ATCC) as measured by qPCR (*n* = 3). (B) *Rankl* mRNA expression in MC3T3 osteoblastic cells treated with ISO, measured by qPCR (*n* = 3). (C) *Sdf1* mRNA expression in BMSCs after ISO treatment, measured by qPCR (*n* = 2). (D and E) Expression of *CXCR4* and *RANK* mRNA after ISO treatment in MDA-231 cells, measured by qPCR (*n* = 2). (F) Cell growth as measured in vitro over 5 d by recording GFP fluorescence, with or without 250 ng/mL rRANKL. Data are plotted as means ± SEM; * *p*<.05; *** *p*<.001.
(TIF)

Figure S4 RANKL increases migration, but not cell growth or *PTHrP* expression. (A) Transwell migration assay in response to rRANKL, 200 ng/mL (*n* = 3). (B) In vitro cell proliferation assays comparing MDA-231 controls cells, Rank^scramble^ cells, or Rank^low^ cells (*n* = 3). (C) *PTHrP* expression as measured by qPCR in MDA-231 control cells treated with rRANKL (*n* = 2). Data are plotted as means ± SEM; ** *p*<.005.
(TIF)

Figure S5 MC3T3 prevents isoproterenol-induced decreased growth in MDA-231 cells. Effects of ISO on MDA-231 cell proliferation when cells were grown on tissue culture plastic (alone control) or on monolayers of osteoblastic MC3T3 or fibroblastic NIH3T3 cells (*n* = 3). Data are plotted as means ± SEM; *** *p*<.001.
(TIF)

Figure S6 Isoproterenol does not affect hemodynamic parameters after 24 h in athymic mice. Time course of isoproterenol effects on heart rate shown in (A) and (B). Systolic (C) and diastolic (D) ventricular volume using ((4/3)*(∏)) *(D/2)^3^ ventricle as a sphere. Aortic parameters of left ventricular outflow tract during systole (E) and aortic diameter (F). Measurements were taken with Visualsonics Vevo770 ultrasound on anesthetized athymic mice (*n* = 3).
(TIF)

## Abbreviations

ALCAM: Activated leukocyte cell adhesion molecule CD166
BMSCs: bone marrow stromal cells
β1/β2AR: beta1/beta2 adrenergic receptor
CIS: chronic immobilization stress, restraint stress
CXCL12: chemokine (C-X-C motif) ligand 12
CXCR4: C-X-C chemokine receptor type 4
GFP: green fluorescent protein
HPA: hypothalamic pituitary adrenal axis
HSC: hematopoietic stem cell
IC: intracardiac
IL-6: interleukin 6
ISO: isoproterenol
MFD: mammary fat pad
NE: norepinephrine
PTHrP: parathyroid hormone-related protein
RANK: Receptor Activator of Nuclear Factor κ B
RANKL: Receptor Activator of Nuclear Factor κ B (Ligand)
SDF-1: stromal derived factor-1
SNS: sympathetic nervous system
uCTI: micro-computed tomography
VEGF: vascular endothelial growth factor
