# Supplementary figures and images for: Stimulation of Host Bone Marrow Stromal Cells by Sympathetic Nerves Promotes Breast Cancer Bone Metastasis in Mice
Source: PLoS Biol. 2012 Jul 17;10(7):e1001363. doi: 10.1371/journal.pbio.1001363 (PMC3398959; doi:10.1371/journal.pbio.1001363)

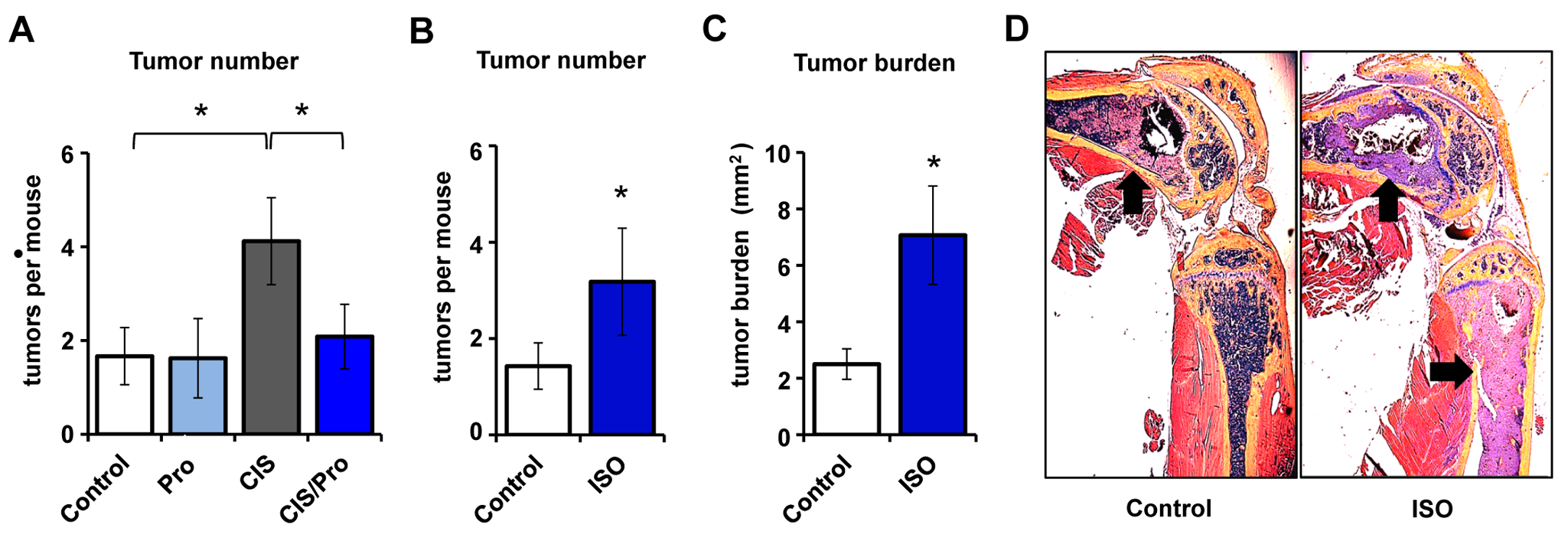

Supplement: Figure S1 — ß2AR stimulation increases total number and growth of bone tumors. Quantification of total number of osseous tumors per mouse (A) with chronic Immobilization Stress (CIS) and/or daily propranolol (Pro) (n = 10) and total number (B) and size (C) of tumors in bone 28 d after tumor inoculation (n = 8). (D) Representative histological images of modified H&E, phloxine, orange G-stained paraffin sections. Tumors (black arrows) are identified by pink staining. Data are plotted as means ± SEM; * p<.05; *** p<.001. (TIF) [file pbio.1001363.s001.tif]

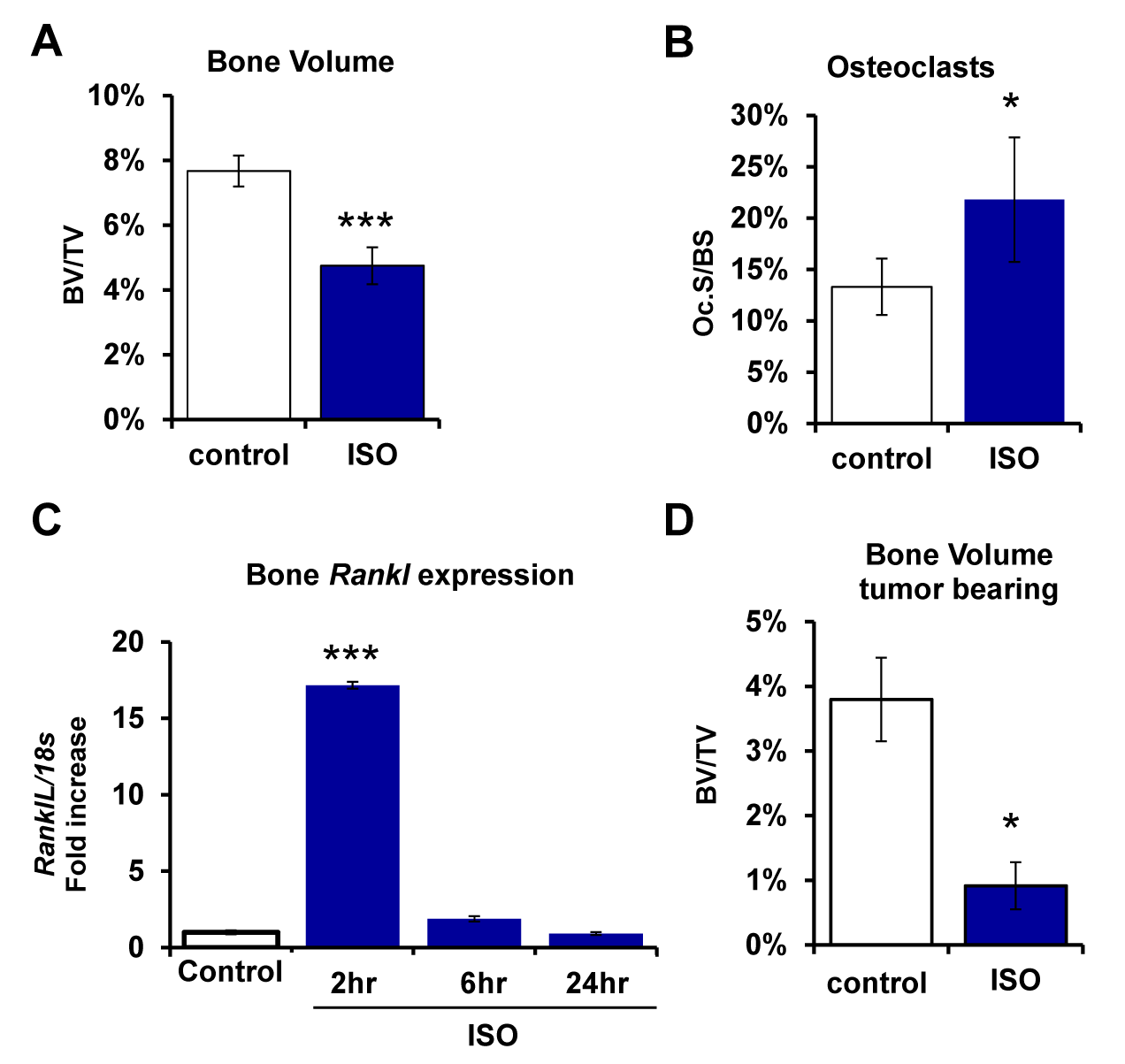

Supplement: Figure S2 — ß2AR stimulation increases bone resorption in athymic nude mice. (A) Tibial Bone Volume/Tissue Volume (BV/TV) of non-tumor bearing athymic mice treated daily with ISO for 4 wk (n = 9). (B) Osteoclast Surface per trabecular Bone Surface (Oc.S/BS) in non-tumor bearing tibiae (n = 9). (C) Bone Rankl mRNA expression assessed by qPCR (n = 4). (D) Tibial BV/TV of tumor-bearing athymic mice treated daily with ISO for 4 wk after intracardiac injection of MDA-231 tumor cells (n = 9). Data are plotted as means ± SEM; * p<.05; *** p<.001. (TIF) [file pbio.1001363.s002.tif]

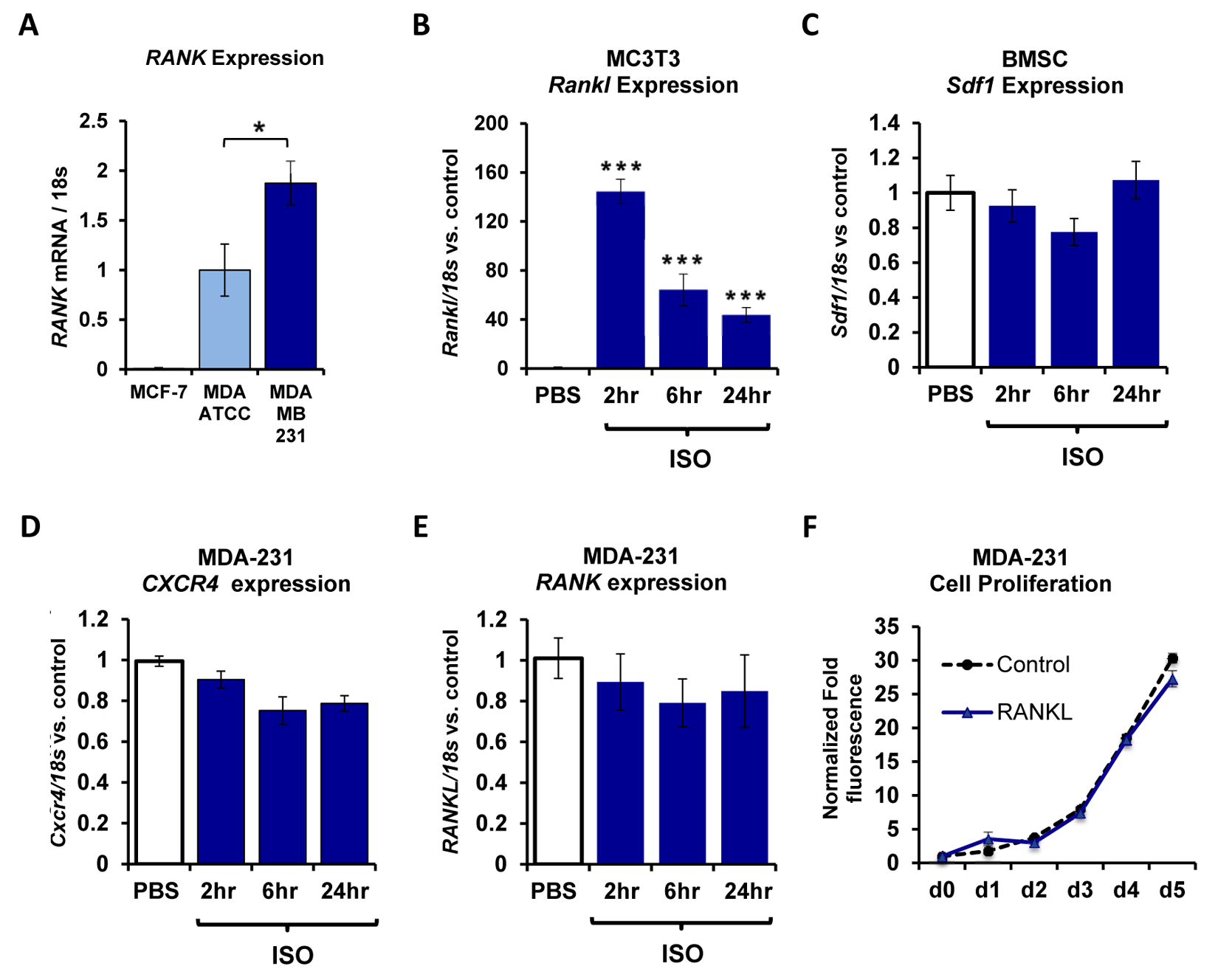

Supplement: Figure S3 — Rankl, but not Sdf1 expression, is increased in osteoblasts upon ß2AR stimulation. (A) RANK mRNA expression in the MDA-231 bone metastatic sub-clone compared to MCF-7 and parental MDA-MB-231 (ATCC) as measured by qPCR (n = 3). (B) Rankl mRNA expression in MC3T3 osteoblastic cells treated with ISO, measured by qPCR (n = 3). (C) Sdf1 mRNA expression in BMSCs after ISO treatment, measured by qPCR (n = 2). (D and E) Expression of CXCR4 and RANK mRNA after ISO treatment in MDA-231 cells, measured by qPCR (n = 2). (F) Cell growth as measured in vitro over 5 d by recording GFP fluorescence, with or without 250 ng/mL rRANKL. Data are plotted as means ± SEM; * p<.05; *** p<.001. (TIF) [file pbio.1001363.s003.tif]

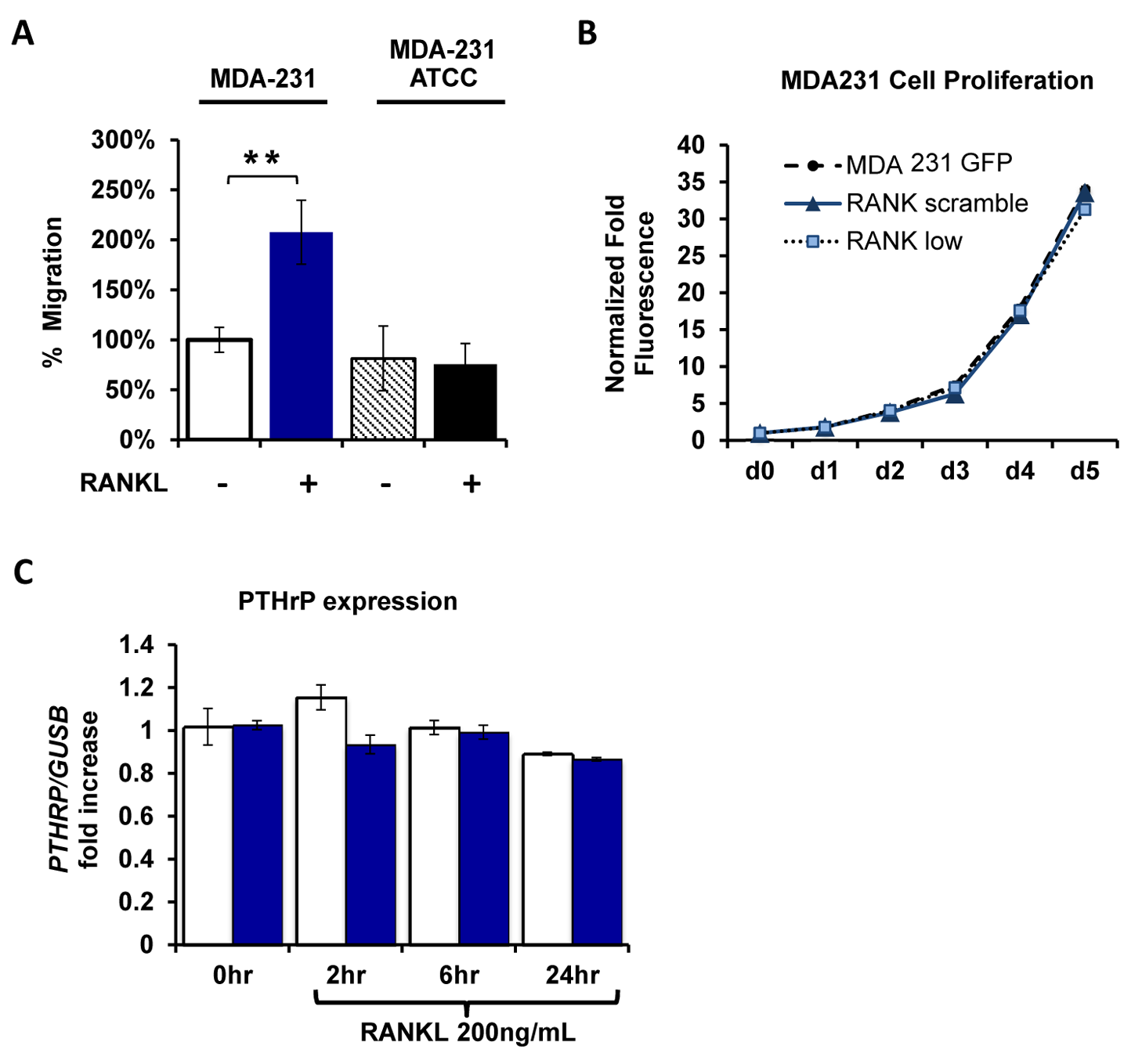

Supplement: Figure S4 — RANKL increases migration, but not cell growth or PTHrP expression. (A) Transwell migration assay in response to rRANKL, 200 ng/mL (n = 3). (B) In vitro cell proliferation assays comparing MDA-231 controls cells, Rankscramble cells, or Ranklow cells (n = 3). (C) PTHrP expression as measured by qPCR in MDA-231 control cells treated with rRANKL (n = 2). Data are plotted as means ± SEM; ** p<.005. (TIF) [file pbio.1001363.s004.tif]

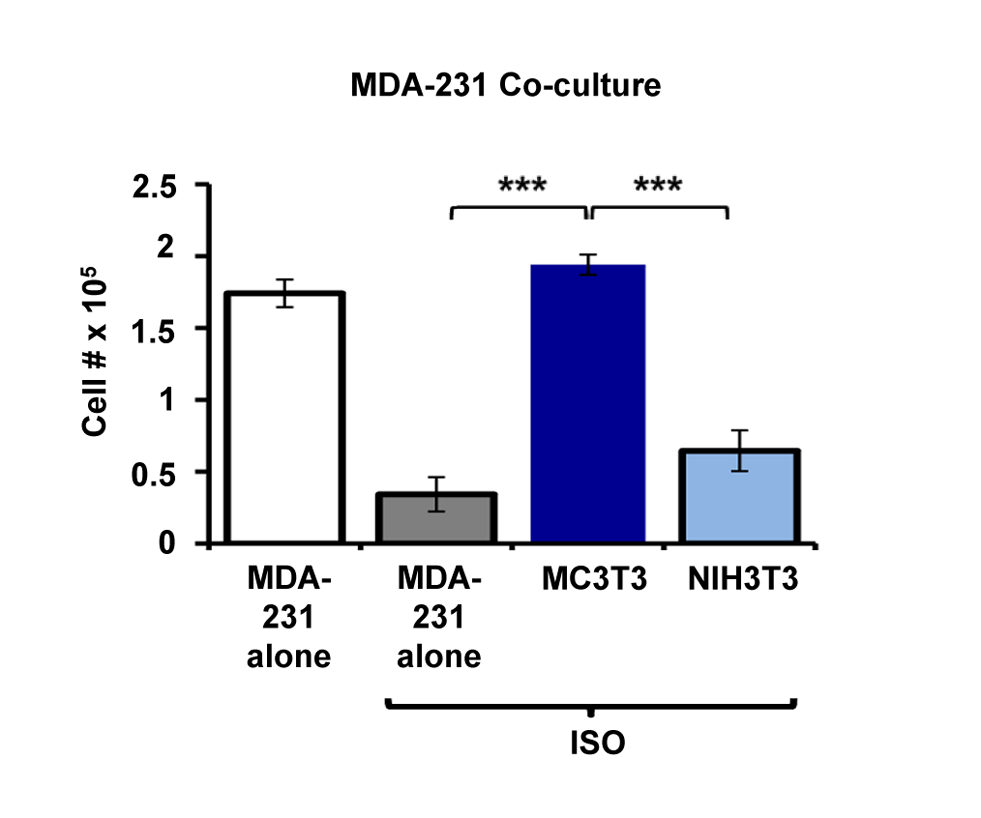

Supplement: Figure S5 — MC3T3 prevents isoproterenol-induced decreased growth in MDA-231 cells. Effects of ISO on MDA-231 cell proliferation when cells were grown on tissue culture plastic (alone control) or on monolayers of osteoblastic MC3T3 or fibroblastic NIH3T3 cells (n = 3). Data are plotted as means ± SEM; *** p<.001. (TIF) [file pbio.1001363.s005.tif]

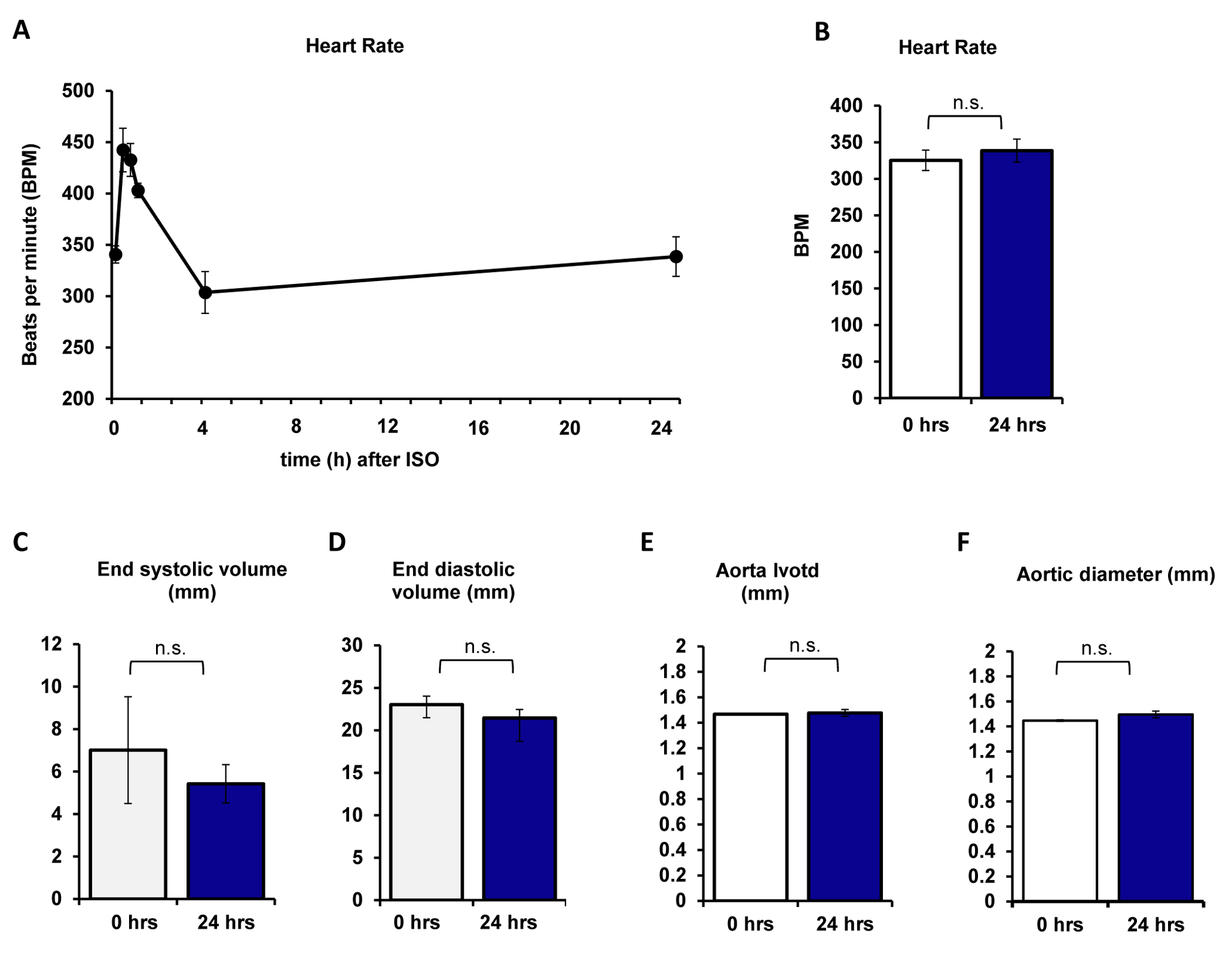

Supplement: Figure S6 — Isoproterenol does not affect hemodynamic parameters after 24 h in athymic mice. Time course of isoproterenol effects on heart rate shown in (A) and (B). Systolic (C) and diastolic (D) ventricular volume using ((4/3)*(∏)) *(D/2)3 ventricle as a sphere. Aortic parameters of left ventricular outflow tract during systole (E) and aortic diameter (F). Measurements were taken with Visualsonics Vevo770 ultrasound on anesthetized athymic mice (n = 3). (TIF) [file pbio.1001363.s006.tif]
